# The South African Regulatory System: Past, Present, and Future

**DOI:** 10.3389/fphar.2018.01407

**Published:** 2018-12-04

**Authors:** Andrea Keyter, Shabir Banoo, Sam Salek, Stuart Walker

**Affiliations:** ^1^Department of Pharmacy, Pharmacology and Postgraduate Medicine, School of Life and Medical Sciences, University of Hertfordshire, Hatfield, United Kingdom; ^2^Department of Health, South African Health Products Regulatory Authority, Pretoria, South Africa; ^3^Department of Pharmacy and Pharmacology, University of the Witwatersrand, Johannesburg, South Africa; ^4^Centre for Innovation in Regulatory Science, London, United Kingdom

**Keywords:** South African Health Products Regulatory Authority (SAHPRA), Medicines Control Council, MCC, legislation, risk-based review

## Abstract

The drive for improved regulatory systems and the establishment of a more effective regulatory framework in South Africa has been evident for the past two decades but despite political intentions and legislative revisions success has been limited to date. Efforts to address the increasing volume of applications that have been received have to date failed and resources have been stretched to capacity resulting in the development of a significant backlog and extended timelines for product registration. The promulgation of the recently amended Medicines and Related Substance Act of 1965 triggered the establishment of the South African Health Products Regulatory Authority (SAHPRA) as a separate juristic person outside of the National Department of Health to replace the former medicine regulatory authority the Medicines Control Council (MCC). The aim of this review is to provide the historical context supporting the new regulatory environment in South Africa and the transition from the MCC to SAHPRA. Key recommendations to SAHPRA to ensure the full potential of the new regulatory environment in South Africa include: establishing a quality management system to safeguard accountability, consistency and transparency and to streamline the implementation of good review practices including quality decision-making practices and benefit-risk assessment; the measurement and monitoring of regulatory performance, targets for overall approval time and key review milestones to instill a culture of accurate metrics collection and measurement of key performance indicators and their continuous improvement and the employment of a risk-based approach to the evaluation of medical products and codify the use of facilitated regulatory pathways in policy and culture. The application of a risk-based approach to regulatory review commensurate with a product’s risk to patients will facilitate the application of increased resources for pharmacovigilance activities and to support the reliance and recognition of reference agencies.

## Introduction

Ensuring effective medicine regulation through the strengthening of regulatory systems and improvement of regulatory performance has become a priority for both NRAs and governments worldwide. With the support of government NRAs are responsible for protecting and promoting public health, implementing rigorous regulatory standards and maintaining an assured supply of medical products that are safe, effective and of good quality (Rägo et al., 2008; Ndomondo-Sigonda et al., 2017; World Health Organization [WHO], 2018). Despite the critical role that NRAs play within national healthcare systems the importance of medical product regulation often goes “under-recognized” and is often “under-funded” (Rägo et al., 2008). The WHO has indicated that “at least 30% of NRAs do not have the capacity to perform core regulatory functions” (Rägo et al., 2008) and “without adequate financial support regulatory authorities do not have the necessary resources to sustain effective regulation of medical products” (World Health Organization [WHO], 2003).

Global trends toward increased pressure on NRAs of all sizes and capacity due to the increased volumes of applications received, the complexity of the submissions and the increased number of categories of medical products have been noted (World Health Organization [WHO], 2014a). These trends and statistics resonate with many NRAs in low- and middle-income countries that have historically been faced with resource constraints (World Health Organization [WHO], 2014b) and that have not participated in global harmonization initiatives or development programs aimed at strengthening regulatory systems (Preston et al., 2012). Efforts to address the challenges faced by NRAs in resource-limited settings have focused on identifying and performing core regulatory functions that have to be undertaken directly by NRAs to meet country or regional needs (Ward, 2014; World Health Organization [WHO], 2014b). NRAs have also been encouraged by the WHO to consider regulatory convergence and to collaborate with and recognize work done by other regulators to ease the regulatory burden (Ward, 2014; World Health Organization [WHO], 2014b).

Resolution WHA67.20 emanating from the Sixty-seventh World Health Assembly in 2014 identified the need for effective regulatory systems and highlighted that “inefficient regulatory systems create barriers for access to safe, effective and quality medical products” (World Health Assembly, 2014). The drive for improved regulatory systems and the establishment of a more effective regulatory framework in South Africa has been evident for the past two decades but despite political intentions and legislative revisions success has been limited to date.

It is suggested that while multi-factorial elements have resulted in a backlog in medicines registration significant pro-access policies compounded by legislative requirements for the expedited review of medicines on the Essential Medicines List, most of which are generics, may be at the root of the problem (Leng et al., 2015). Efforts to address the increasing volume of applications that have been received have to date failed and resources have been stretched to capacity resulting in the development of a significant backlog and extended timelines for product registration. The median approval times for fast track applications approved by the MCC in 2015, 2016, and 2017 were 1218, 921, and 609 calendar days, respectively (Keyter et al., 2018). There was no target time set for the overall review time of NCEs and the median approval times for NCE marketing authorization applications approved in 2015, 2016, and 2017 were 1161, 1678, and 1422 calendar days, respectively (Keyter et al., 2018). These data demonstrate that the MCC was not able to achieve the target timelines of 250 calendar days set for fast track applications nor meet the targets in 2015, 2016, and 2017 for the key milestones within the regulatory review process (Keyter et al., 2018).

Pharmaceutical companies, private clinical research organizations, academic clinical research groups and civil society organizations have complained that delays and the backlog in medicines registration were harming patients’ access to affordable medicines (Leng et al., 2015). “Prior to 2005 the number of applications received and the number of registration certificates issued were in equilibrium, however, from 2005 the number of applications submitted more than doubled whereas the number of certificates issued remained approximately the same” (Leng et al., 2015). The South African NRA has a historical average of receiving approximately 4700 applications per year but has demonstrated that it can only process approximately 2550 applications per annum (South African Health Products Regulatory Authority [SAHPRA], 2018). SAHPRA has inherited a backlog of approximately 16 000 applications that includes all applications submitted up to 31 January 2018 which are yet to receive final approval (South African Health Products Regulatory Authority [SAHPRA], 2018). The SAHPRA Board aims to clear the backlog within the next 2 years and with more than half of new registration applications being at least 5 years old industry has been requested to “opt-in” for applications submitted in 2013 or earlier. Submissions within the backlog need to be consolidated, updated and resubmitted to ensure that submissions requiring evaluation reflect current data (South African Health Products Regulatory Authority [SAHPRA], 2018). Applications will be segmented and prioritized according to public health priorities (South African Health Products Regulatory Authority [SAHPRA], 2018). SAHPRA will operationalize reliance models for product review supported by optimal staffing solutions, implementation of a digitally powered approach to evaluation, effective change management and improved transparency and accountability (South African Health Products Regulatory Authority [SAHPRA], 2018).

The promulgation of the recently amended Medicines and Related Substance Act, 1965 (Act 101 of 1965) hereafter referred to as the Medicines Act triggered the establishment of the SAHPRA as a separate juristic person outside of the National Department of Health to replace the former medicine regulatory authority the MCC. The amended Medicines Act saw the scope of the Authority’s mandate extended to make provision for the regulatory oversight of medical devices and complementary medicines in South Africa and to make provision for the Authority to establish and strengthen collaborative initiatives with any other regulatory authorities or institutions (Republic of South Africa, 2017).

The aim of this review is to provide the historical context supporting the new regulatory environment in South Africa and the transition from the MCC to SAHPRA.

## The Medicines Control Council

Prior to the establishment of SAHPRA in February 2018 the MCC was the national medicines regulatory authority of South Africa responsible in terms of the Act to provide for the monitoring, evaluation, regulation, investigation, inspection, registration and control of human and veterinary medicines, scheduled substances, clinical trials and related matters in the public interest. The statutory obligations of the MCC were to ensure that medicines that were available in South Africa met the required standards of quality, safety, and efficacy (Medicines Control Council [MCC], 2006).

### Organizational Structure

The MCC was a statutory body appointed by the Minister of Health consisting of not more than 24 members including the chairs of the expert committees. In addition the council appointed external experts to serve on various expert committees overseeing medicine registration, regulation and control functions. Overall there were 11 active expert committees including the Biological Medicines, Clinical, Clinical Trials, Complementary Medicines, GxP, Legal, Medical Devices, Names & Scheduling, Pharmaceutical & Analytical, Pharmacovigilance and Veterinary Clinical Committees (Medicines Control Council [MCC], 2017). The skills of the members of council and its committees were written into law and included expertise in toxicology and medicine safety, basic and clinical pharmacology, biotechnology, pharmaceutics, internal medicine, virology, pharmaceutical chemistry, neonatology, pediatrics, immunology, veterinary science, complementary medicines and law (Medicines Control Council [MCC], 2017).

**FIGURE 1:**
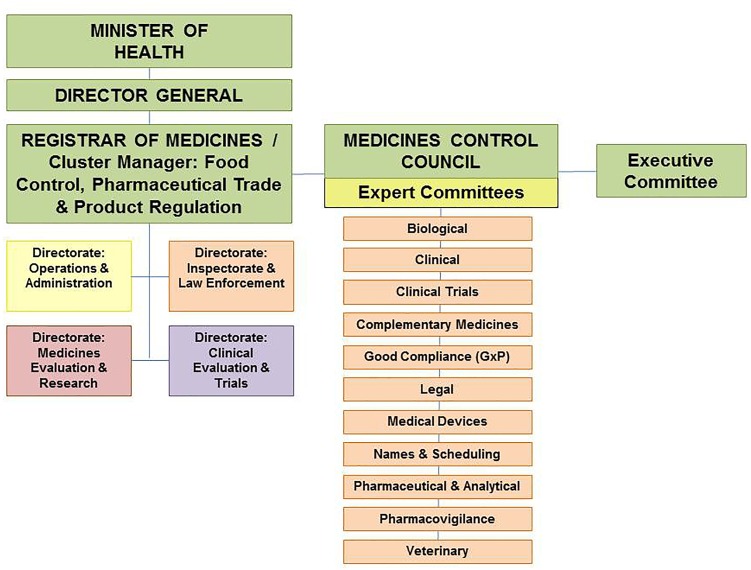
Organizational structure of the Medicines Control Council.

The Office of the Registrar served as the Executive Secretary to the MCC and provided administrative and technical support to Council and its activities. The Office of the Registrar was a Chief Directorate within the National Department of Health known as the Cluster: Food Control, Pharmaceutical Trade and Product Regulation. There were four Directorates within the Cluster namely Operations and Administration, Inspectorate and Law Enforcement, Medicines Evaluation and Research and Clinical Evaluation and Trials. The staff complement of the Cluster included doctors, pharmacists, veterinarians, scientists and administrative staff (Medicines Control Council [MCC], 2017). The MCC organizational structure is depicted in Figure 1 (South African Health Products Regulatory Authority [SAHPRA], 2016).

### Regulatory Review Process

**FIGURE 2:**
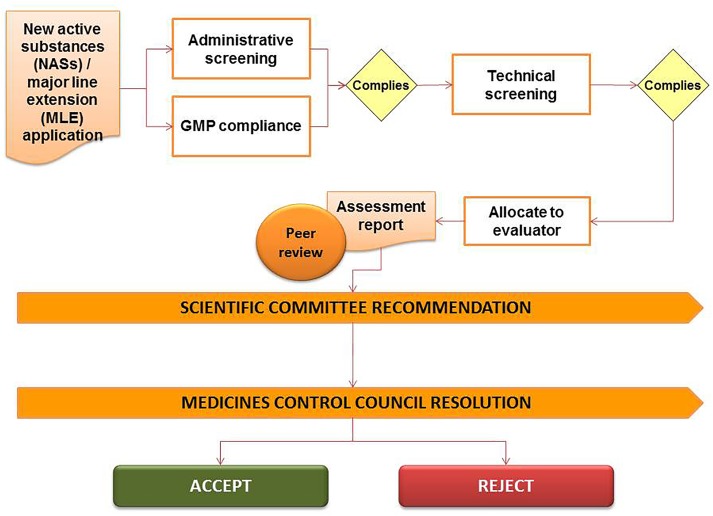
Regulatory review process of the Medicines Control Council.

The registration of medicines in South Africa is governed by the provisions and requirements of the Medicines Act including the regulations and the published guidelines. Legislative frameworks require that medicines including NCEs, multisource/generic medicines, biological medicines, complementary medicines and veterinary medicines are evaluated by the NRA prior to marketing of the product. Applicants are required to submit technical dossiers to demonstrate the quality, safety, and efficacy of such medicines intended for sale in South Africa. The confidentiality of information submitted to the NRA is governed by Section 34 of the Medicines Act regarding the preservation of secrecy. The regulatory review process of the MCC is presented in Figure 2 and provides a simple representation of the review and authorization of applications that are approved in the regulatory review cycle.

The NRA made use of both internal and external expertise to evaluate applications for the registration of medicines. A full review of the safety, quality, and efficacy data, together with the assessment reports prepared by reviewers were considered by the various expert committees to make recommendations on the approval of the proprietary name of the product, the allocation of a scheduling status for the active pharmaceutical ingredient and the evaluation of the GMP status of the applicant, the manufacturer of the active pharmaceutical ingredient, the manufacturer of the finished pharmaceutical product, the packer and the quality control laboratory. The final decision for authorization or refusal was made by the MCC.

### History of Enabling Legislation

The introduction of the regulation of medicines in South Africa was initiated in the 1960s when the National Department of Health appointed the Snyman Commission to investigate the high cost of medicines and medical services in South Africa (Snyman, 1965). The report of the Commission of Inquiry recommended at the time that medicines be controlled in terms of their “purity, safety and therapeutic efficacy” (Gouws, 2003). These recommendations resulted in the promulgation of the Drugs Control Act, 1965 (Act 101 of 1965) and the establishment of the Drugs Control Council responsible for the control of medicines for human use (Gouws, 2003). The introduction of a registration procedure in 1968 meant that all medicines intended for sale in South Africa were evaluated and approved by the Drugs Control Council prior to entering the market. Medicines available on the market prior to 1968 were initially exempt from these requirements and were referred to as “old medicines” (Gouws, 2003). Over the next three decades the legislative framework and regulatory requirements were amended several times to reflect the intentions of the regulatory authority as it strived toward improved control of medicines in South Africa. Some of the important amendments made to the principal Act, the Medicines and Related Substances Act, 1965 (Act 101 of 1965) are listed in Table 1 (Gouws, 2003) and the historic projects and legislative changes are noted in Table 2.

**Table 1:** Amendments to Drug Control Act 1965.

Amendment Number	Change
Amendment Act No 29 of 1968	Drugs that were subjected to registration were defined
	Categories for the classification of these drugs were defined
Amendment Act No 88 of 1970 Amendment Act No 95 of 1971	Made provision for the control of advertising of drugs.
Amendment Act No 65 of 1974	The term “drug” was replaced with “medicine”
	The Drugs Control Council was changed to the Medicines Control
	Council
	The constitution of the Medicines Control Council, remuneration of the Council members and the appointment of the Committees of Council and a Medicines Control Appeal Board was defined.
Amendment Act No 17 of 1979	The mandate of the Act was extended to include the regulatory oversight of veterinary medicines, including the registration, labeling and advertising thereof.
Amendment Act No 94 of 1991	The powers, functions and constitution of the Council were defined
	The establishment of the Medicines Control Appeal Board was repealed
	Provisions for an alternative appeal procedure against the decision of the Council were defined.
Amendment Act No 90 of 1997	The MCC was established as a juristic person
	Members of the Council or the Committees were required to declare commercial interests related to the pharmaceutical or health care industry
	The members of the Executive Committee of the Council, were to be appointed subject to the approval by the Minister of Health
	Conditions prohibiting the sale of any medicine, which were subject to registration, and which were not registered, were defined
	Provision for expedited registration of essential medicines
	Re-registration of medicines every 5 years
	Provisions for compulsory licensing and parallel importation
	Provisions to enable generic substitution were defined
	A Pricing Committee for medicines was established
	The process of appeal against a decision of the Director-General of Health was defined
	Provision was made for acquiring of additional funds by the Council
	The powers of the Minister of Health to make regulations pertaining to the Medicines Act were further defined.
Amendment Act 59 of 2002	Provision was made for the appointment of Deputy Registrars
	the term of office of the Pricing Committee members was defined
	Regulations relating to the marketing of medicines was defined and
	Repeal of the SAMMDRA Act.

**Table 2:** Historic Projects and Legislative Changes.

Timeline	Initiated by	Project Team	Objective	Recommendation	Result
1960	South African National Department of Health	Snyman Commission	• Investigate the high cost of medicines and medical services in South Africa	• Medicines should be controlled in terms of their “purity, safety and therapeutic efficacy”	• Promulgation of the Drugs Control Act, 1965 (Act 101 of 1965)
					• Establishment of the Drugs Control Council
1998	Minister of Health, Nkosazana Dlamini-Zuma	Advisory Panel	• Review the medicine regulatory environment in South Africa	• Endorsed the restructuring of the MCC with the aim of improving operational efficiencies	• The new Amendment Act establishing the South African Medicines and Medical Devices Regulatory Authority (SAMMDRA) to replace the MCC was passed by Parliament
2007	Minister of Health, Manto Tshabalala-Msimang.	Ministerial Task Team led by Professor Green-Thompson	• Report on the restructuring of the MCC	• The establishment of a new NRA to replace the MCC referred to as SAHPRA	• Further amendment of the principal Act
				• The need for international and regional harmonization	• The Medicines Amendment Act, 2008 was signed into law by then President Kgalema Motlanthe in 2009 but not implemented
				• The need for collection of metrics to facilitate the measurement and monitoring of regulatory performance	
2009	Minister of Health, Barbara Hogan	Project team led by Dr Nicholas Crisp	• Revive legislative endeavors directed toward regulatory reform	• Develop the business case for SAHPRA	• Further amendment to the Medicines Amendment Act, 2008
			• Establishment of an improved NRA	• Identification of further legislative amendments	• The Medicines and Related Substances Amendment Bill, 2012 was published for comment in March 2012
2012	Director General of Health, Malebona Precious Matsoso,	Health Products Technical Task Team (HPTTT)	• Advise on the key legislative, programmatic, infrastructural, structural and operational elements required for the transition to SAHPRA	• Benchmark regulatory procedures in identified technical and operational areas	• Finalization of the Medicines and Related Substances Amendment Bill, 2012
				• Explore mechanisms for information sharing and systems to establish mutual recognition for registration requirements and product approval	• The new Medicines Amendment Act, 2015 was approved (January 2016)
					• The draft SAHPRA business case prepared by Dr Nicolas Crisp was amended to reflect current developments and the key elements required for the transition of the MCC to SAHPRA

The Amendment Act, 1997 (Act 90 of 1997) was the first legislative amendment to be made to the principal Act following the change of government in South Africa after the general elections held in 1994 (Gouws, 2003). With this change came the adoption of a program for health reform and the launch of the National Drug Policy (Gouws, 2003). The Amendment Act, 1997 (Act 90 of 1997) was promulgated in 1997 and Section 15C specifically was the subject of a legal challenge by the PMA which prevented the implementation of the Amendment Act, 1997 (Act 90 of 1997) until 2003 (Pharmaceutical Manufacturers Association [PMA], 1998). The then Minister of Health, Nkosazana Dlamini-Zuma appointed an advisory panel to review the medicine regulatory environment in South Africa (Dukes et al., 1998). In December 1998 a report titled “Operational and Financial Review - Discussion Draft” prepared by KPMG also endorsed the restructuring of the MCC with the aim of improving operational efficiencies. On the recommendation of the ministerial advisory panel a new Amendment Act (Republic of South Africa, 1998) establishing the SAMMDRA to replace the MCC was passed by Parliament. The SAMMDRA Act was promulgated prematurely without the necessary Regulations and was subsequently set aside (Pharmaceutical Manufacturers Association [PMA], 2000).

In late 2007 yet another decision was taken to restructure the MCC by establishing a new authority as a public entity outside of the National Department of Health. A report on the restructuring of the MCC was presented by a Ministerial Task Team led by Professor Green-Thompson who was appointed as a Special Advisor to the Minister of Health, Manto Tshabalala-Msimang. (South African Health Products Regulatory Authority [SAHPRA], 2016). The Green-Thompson Report recommended the establishment of a new NRA to replace the MCC referred to as SAHPRA and emphasized the need for international and regional harmonization to support reliance and recognition frameworks with other regulatory authorities (Green Thompson, 2008). The report amongst others recommended extending the regulatory mandate of the authority to include medical devices and highlighted the need to effect benefit-risk assessment of medicines and quality decision-making principles to support transparent regulatory decision-making (Green Thompson, 2008). Regulatory models of other NRAs were benchmarked and a key recommendation from this report informed the need for collection of metrics to facilitate the measurement and monitoring of regulatory performance and the impact of the proposed changes to the regulatory review process (Green Thompson, 2008). The recommendations of the Green-Thompson report resulted in a further amendment of the principal Act and the Medicines Amendment Act, 2008 (Act 72 of 2008) was signed into law by then President Kgalema Motlanthe in 2009 but not implemented (South African Health Products Regulatory Authority [SAHPRA], 2016). The reason for this was multi-factorial and included the need for strengthened governance and certain transitional provisions.

A project team led by Dr Nicholas Crisp was appointed in 2009 by the Minister of Health, Barbara Hogan to revive legislative endeavors directed toward regulatory reform and the establishment of an improved NRA (South African Health Products Regulatory Authority [SAHPRA], 2016). The remit of this project team was to develop the business case for SAHPRA as well as the transitional mechanisms and the identification of further legislative amendments. Through the work of the project team further amendments were made to the Medicines Amendment Act, 2008 (Act 72 of 2008) and the Medicines and Related Substances Amendment Bill, 2012 was published for comment in March 2012 (South African Health Products Regulatory Authority [SAHPRA], 2016). In July 2012 the project team presented a draft business case for the establishment of SAHPRA (South African Health Products Regulatory Authority [SAHPRA], 2012). The business case put forward a motion to establish SAHPRA as a Schedule 3A Public Entity to reinforce the political will to establish an NRA with operational autonomy and accountability. The business case defined an extended mandate for SAHPRA including the regulatory oversight of food, complementary medicines, medical devices and radiation control. The report demonstrated historical under-funding of the NRA linked with recommendations for levying increased fees and motivated for “proactive remuneration strategies” to attract and retain the expertise required to execute the mandate of SAHPRA. It also expanded on the over-reliance on paper-driven systems and the necessity for an EDMS (South African Health Products Regulatory Authority [SAHPRA], 2012).

The Director General of Health, Malebona Precious Matsoso, also appointed a Health Products Technical Task Team (HPTTT) in 2012 to consider the project team’s recommendations and to advise further on the key legislative, programmatic, infrastructural, structural and operational elements required for the transition to SAHPRA (Health Products Technical Task Team, 2014; Pharasi and Banoo, 2015). The HPTTT as part of its mandate engaged several NRAs (the European Medicines Agency, U.S. Food and Drug Administration, Swissmedic, the United Kingdom Medicines and Healthcare Products Regulatory Agency and Australian Therapeutic Goods Administration) to examine and benchmark regulatory procedures in identified technical and operational areas as well as to explore mechanisms for information sharing and systems to establish mutual recognition for registration requirements and product approval. These activities were also aimed at maximizing regulatory capacity and operations under SAHPRA through understanding the structure and functioning of these agencies in line with international best practice standards. One of the outcomes of the HPTTT work was the finalization of the Medicines and Related Substances Amendment Bill, 2012 and its introduction to Parliament for consideration. The new Medicines Amendment Act, 2015 (Act 14 of 2015) was approved by the Parliament, assented to by the President in December 2015 and published in the *Government Gazette* in January 2016 (South African Health Products Regulatory Authority [SAHPRA], 2016). The draft SAHPRA business case prepared by Dr Nicolas Crisp was further amended by the HPTTT to reflect current developments and the key elements required for the transition of the MCC to SAHPRA (South African Health Products Regulatory Authority [SAHPRA], 2016). The amended business case defined the preparation and operationalization of the transition, directed the development of a new fee schedule published in September 2015 to support the viability of the new NRA, informed the development and publication of the regulations for medical devices in December 2016 and confirmed the withdrawal of food control from the regulatory ambit of SAHPRA (South African Health Products Regulatory Authority [SAHPRA], 2016). With the focus on financial and operational considerations these transitional arrangements overlooked the critical need for the review and improvement of the regulatory review process of the NRA as recommended in the Green-Thompson report. On the 1st June 2017 the amendments to the principal Act were enacted via proclamation of the Medicines and Related Substances Amendment Act, 2008 (Act 72 of 2008) read together with the Medicines and Related Substances Amendment Act, 2015 (Act 14 of 2015).

## The South African Health Products Regulatory Authority

In February 2017 SAHPRA was legally established as a Schedule 3A Public Entity in terms of the PFMA, 1999 (Act 1 of 1999) to fulfill specific responsibilities on behalf of national government (National Treasury, 2015). As a Schedule 3A Public Entity SAHPRA is separate juristic person outside of the National Department of Health accountable for sound corporate governance practices and adherence to compliance codes in terms of relevant legislation, financial regulations, directives, policies and procedures (National Treasury, 2015).

**FIGURE 3:**
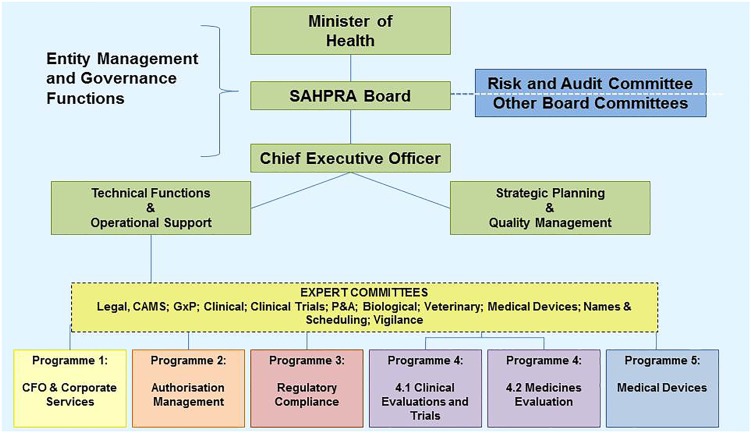
Transitional organizational structure of the South African Health Products Regulatory Authority.

In October 2017 the Minister of Health, Aaron Motsoaledi, announced the appointment of 15 SAHPRA Board members. The Board members are appointed to serve for a period of 3 years under the leadership of Professor Helen Rees, the outgoing Chairperson of the MCC and the first Chairperson of the SAHPRA Board. In contrast to the MCC the SAHPRA Board has full operational autonomy and accountability. Through the Board the Authority is accountable to the Minister of Health (Republic of South Africa, 2017). The SAHPRA Board after consultation with the Minister of Health must appoint a suitably qualified person as the CEO of the Authority (Republic of South Africa, 2017). The CEO is accountable to and reports to the SAHPRA Board and is responsible for the general administration of the Authority and for the carrying out of any functions assigned to the Authority (Republic of South Africa, 2017). The organizational structure of SAHPRA is depicted in Figure 3 (South African Health Products Regulatory Authority [SAHPRA], 2016). The four Directorates depicted in Figure 1 will be replaced by five programs responsible for performing the regulatory activities of the Authority. In order to ensure continuity transitional arrangements have been put in place for the expert committees to continue providing scientific expertise and support. A Regulatory Advisory/Oversight Committee for medicines and medical devices has been appointed by the CEO in consultation with the SAHPRA Board to investigate and report to the Authority on any matter within its purview in terms of Medicines and Related Substances Act, 1965 (Act 101 of 1965). The SAHPRA Board may appoint one or more committees from among its members to assist it with the performance of its functions and has appointed a TORS Committee with investigation into the backlog in application for registrations as part of its remit.

“The legislative mandate of SAHPRA is derived from the Constitution of the Republic of South Africa, 1996 which places obligations on the state to progressively realize socio-economic rights including access to health care as well as the National Health Act, 2003 (Act 61) and the Medicines and Related Substances Act, 1965 (Act 101 of 1965). According to the Medicines and Related Substances Act, 1965 (Act 101 of 1965), “SAHPRA’s obligations include ensuring public protection, ensuring transparency and accountability in its operations and being responsive to the regulatory environment” (South African Health Products Regulatory Authority [SAHPRA], 2016).

The functions of the Authority are defined in Section 2B of the Medicines and Related Substances Act, 1965 (Act 101 of 1965). The Authority must, in order to achieve its objectives, ensure that the:

• “Evaluation or assessment and registration of medicines and medical devices, is efficient, effective and ethical and that registered medical products meet the defined standards of quality, safety, efficacy, and performance;
• Process of evaluating or assessing and registering medicines and medical devices is transparent, fair, objective and concluded timeously;
• Medicines and medical devices are re-evaluated or reassessed and monitored periodically;
• Existing and new adverse events, interactions and information with regard to post-marketing surveillance and vigilance are monitored, analyzed and acted upon;
• Compliance with existing legislation is being promoted and controlled through a process of active inspection and investigation; and
• Clinical trial protocols are assessed according to prescribed ethical and professional criteria and defined standards” (Republic of South Africa, 2017).

Political will and leadership have seen the efforts for an improved regulatory landscape in South Africa come to fruition as the evolving NRA strives toward an effective and efficient regulatory authority. The key operational differences between the MCC and SAHPRA are highlighted in Table 3. The mandate of SAHPRA has been extended to include medical devices and complementary medicines and the legislative framework for reliance and recognition has been finalized. It is anticipated that improvements to the other operational elements listed in Table 3 will be realized with the establishment of SAHPRA.

**Table 3:** Key Operational differences between Medicines Control Council and South African Health Products Regulatory Authority.

Operational element	MCC	SAHPRA
Mandate	Human and Veterinary Medicines	Medical Devices and Complementary Medicines included
Organizational structure	Under-resourced:	Fully resourced:
	Outsourced expertise	In-house capacity
Harmonization initiatives	Limited scope for reliance mechanisms	Legal framework for reliance mechanisms
Quality management system	Informal implementation of QMS	Formal implementation of QMS
Document management System	Paper-driven	Electronic Document Management Systems-driven
Fee structure	Collection of fees by National Treasury	Retention of user-fees
Service delivery	History of backlogs	Improved timeliness
Stakeholder relationships	Stretched industry relationships	Transparency and accountability

### Extended Mandate

In the past the MCC was mandated to ensure regulatory oversight of human and veterinary medicines. With the promulgation of the amendments to the principal Act the mandate of the Authority has been extended to include medical devices, ionizing and non-ionizing radiation emitting devices, radioactive nuclides and complementary medicines.

### Challenges and Changes

Historically the MCC faced resource constraints as workloads placed on the regulator steadily increased. As a result the MCC became dependent on over-committed external expertise. Evaluation structures which relied on external evaluators lacked effective performance management contracts and did not provide a sustainable mechanism for timely submission of evaluation reports. The regulatory functions mandated to SAHPRA are people-dependent (South African Health Products Regulatory Authority [SAHPRA], 2016). Adequate, competent and motivated human capital plays a vital role in ensuring organizational success (South African Health Products Regulatory Authority [SAHPRA], 2016) “It is the intended goal of SAHPRA to have an adequate number of staff with the right skills mix, at the right level, available and employed in appropriate positions within the organization” (South African Health Products Regulatory Authority [SAHPRA], 2016). Efforts to reform organizational structures within SAHPRA should be prioritized to build and retain in-house scientific skills in order to decrease over-reliance on external expertise.

### Harmonization Initiatives

As an Authority mindful of limited resources and capacity constraints the MCC had always recognized the value of harmonization initiatives and had explored the possibility of implementing reliance mechanisms. In the past the MCC participated in regional collaboration initiatives such as the ZaZiBoNa collaborative work-sharing process which aims to harmonize regulatory efforts between regional NRAs. Harmonization efforts may now be actively enforced as the inclusion of Section 2B(2)(a) and 2B(2)(b) in the Medicines Act provides a mandate for the Authority to liaise with and enter into agreements with any other regulatory authorities or institutions (Republic of South Africa, 2017).

The advantages of such regulatory relationships are offset by a number of prerequisites including the assumption that SAHPRA adopts internationally harmonized guidelines and standards (South African Health Products Regulatory Authority [SAHPRA], 2016), relevant memoranda of understanding and confidentiality agreements are in place with reliable regulatory authorities recognized by SAHPRA (Green Thompson, 2008), that SAHPRA remains accountable for the health and safety of the citizens of South Africa (South African Health Products Regulatory Authority [SAHPRA], 2016), that some regulatory decisions may be made based on the regulatory activities and/or decisions made by other reliable authorities and recognized by SAHPRA (South African Health Products Regulatory Authority [SAHPRA], 2016) and that enhancing regulatory convergence and participating in collaboration and work-sharing initiatives will contribute toward a decreased regulatory burden and a decreased workload on SAHPRA. SAHPRA will also have the opportunity to make better use of the limited resources available to improve post-marketing surveillance activities and will contribute toward efforts to minimize duplication of regulatory efforts (World Health Organization [WHO], 2003).

### Quality Management System

The MCC has recognized the importance of formally implementing quality measures throughout the agency in order to ensure consistency, increase transparency and improve efficiencies. In the past the MCC did not have a dedicated Quality Management Unit, however, contingencies have been put in place to establish such a unit. This unit will be responsible for formalizing the implementation of the QMS for the authority and for performing internal quality audits and for implementing strategies geared for continuous improvement. The implementation of a formalized QMS will ensure that GRevPs are codified into policies and guidelines, regularly monitored and subject to continuous improvement (World Health Organization [WHO], 2016). Through the application of a robust QMS underpinned by the drive to cultivate an integral quality culture the regulatory performance and responsiveness of SAHPRA will be enhanced.

### Document Management System

“A regulatory authority must have an effective system of tracking application assessment processes and decision-making; these systems require an appropriate use of information technology” (Hill and Johnson, 2004). The development of an integrated information system, improvement of the current ICT infrastructure and the use of an EDMS will be essential for SAHPRA. Given the large volume of complex applications submitted to the Authority and the need for optimal document management it is critical that the Authority moves away from the historically paper-driven processes of the MCC. It is the intention of SAHPRA to implement an EDMS that can replace the legacy systems currently in use. SIAMED, a software program adopted from the WHO, is one such system that is used to track and manage applications for registration of medicines. This system has become outdated and will be phased out as electronic systems capable of facilitating the electronic submission of applications and robust document management functionalities are introduced.

### Fee Structure

The historical integration of the MCC into the operations of the South African National Department of Health has not served the MCC well as it worked toward ambitious goals of improved regulatory performance without the financial support required to establish a new regulatory authority that would be a viable regulator of medical products, trusted and respected by the pharmaceutical industry, civil society and patients of the Republic (South African Health Products Regulatory Authority [SAHPRA], 2016). The Act makes provision for the Authority to levy fees for services rendered for example, a fee may be charged for the evaluation and registration of medical products. Fee structures vary significantly between different regulatory authorities. Fees may be set arbitrarily, they may be related to the cost of providing a service or they may be scaled, commensurate with the amount of data submitted and the time required for evaluation of the data.

As SAHPRA develops its organization it is important to consider its fee structure for its future sustainability. In view of the current backlog and protracted review times it is vital that there should be an increase in the resources (e.g., trained staff and infrastructure) to ensure that the new authority achieve its goal in bringing the review times in line with other mature established authorities. Antrobus and Egerton-Warburton (2016) have reported comparative figures on regulatory fees which dramatically illustrate how South Africa fees lags behind several comparable regulatory authorities and they have proposed how to achieve financial sustainability with a carefully revised fee structure. There are, of course, recent examples such as China and Japan who have demonstrated that measured increase in fees has translated into several fold increase in trained staff and subsequently led to reduction in timelines (Antrobus and Egerton-Warburton, 2016; Bujar et al., 2018).

The establishment of SAHPRA as a 3A Public Entity allows for change in that the finances generated by the Authority will be retained. This revenue structure is different to the past model that existed within the MCC whereby incoming fees were collected by the National Treasury and channeled to central government revenue. Although the Authority will be partially funded from the national government funds a key deliverable for SAHPRA will be to raise the required revenue to make the Authority sustainable (South African Health Products Regulatory Authority [SAHPRA], 2016). Suggestions to increase the fees for services levied by the Authority may be a solution but this will require significant improvements in regulatory efficiencies in order to appease the demands and expectations of stakeholders. Furthermore, an opportunity exists to generate more fees as the mandate of the Authority is extended to include the regulation of medical devices, complementary medicines and radiation control (South African Health Products Regulatory Authority [SAHPRA], 2016).

### Service Delivery and Stakeholder Relationships

“SAHPRA has an obligation to effectively implement a regulatory framework that supports regulatory functions, enables the objectives of the National Drug Policy and promotes the priority goals of the National Department of Health” (South African Health Products Regulatory Authority [SAHPRA], 2016). In order to do so it is necessary to improve structures within the Authority and advance the functions of the Authority to develop an accessible regulatory service footprint (South African Health Products Regulatory Authority [SAHPRA], 2016).

Recognition of SAHPRA as a sustainable-well functioning regulatory system is a key feature of the strategic outcome oriented goals for the Authority (South African Health Products Regulatory Authority [SAHPRA], 2016). The effectiveness of the regulatory systems developed, implemented and maintained by SAHPRA must be periodically measured against GRevP and pre-defined performance-based indicators (World Health Organization [WHO], 2014b; South African Health Products Regulatory Authority [SAHPRA], 2016). Global benchmarking of the Authority against the indicators of the global benchmarking tool developed by the WHO to evaluate and grade the maturity level of the regulatory systems of NRAs will also provide a measurement of the Authority’s performance in assuring independent and competent oversight of medical products in South Africa (World Health Organization [WHO], 2017). Delivering on such regulatory performance objectives will also provide a platform for building strong and sustainable relationships with stakeholders with an emphasis on customer satisfaction.

## The Regulatory Review Process in South Africa: Modalities for Change

Through the amendment of the Medicines Act and the establishment of SAHPRA a new era has dawned bringing about new opportunities for regulatory reform and the possibility to re-engineer outdated processes. Priority should be given to addressing the inefficiencies of the current regulatory review process through consideration of different types of product review assessments used by NRAs worldwide in the review of applications for registration of medicines namely the verification review (type 1), an abridged review (type 2) and a full review (type 3) (McAuslane et al., 2009). SAHPRA may decide to continue with the current approach used historically by the MCC whereby a type 3 full independent assessment of quality, efficacy, and safety data is performed in the review of all applications for registration, however, it may be prudent to consider applying a risk-based assessment for those applications already reviewed by reference agencies in order to ensure timely access of medicines and medical devices.

### Risk-Based Approach to the Evaluation of Medicines

Management of limited resources may be improved through the application of a risk-based approach to medical product regulation. This approach allows regulators to direct the appropriate resources required to those medical products that pose a greater risk to patients. The amount of resources applied by the regulator should be commensurate with the level of risk of a medical product and should be applied only to the extent necessary to ensure patient safety (Therapeutic Goods Administration [TGA], 2018). Many NRAs including resourced and mature regulatory authorities make use of FRPs for the assessment of applications for registration of medicines (Liberti, 2018). Primary FRPs are used to decrease review times of medicines that have not been reviewed by another NRA and that are not dependent on the review/decision made by another NRA for example products for unmet needs and oncology (Liberti, 2018). Secondary FRPs are used by NRAs to decrease review times of medicines that have been reviewed by another recognized NRA (Liberti, 2018). The regulatory decision can be expedited through reliance on or recognition of a prior review/decision by another NRA (Liberti, 2018). FRPs inform risk-stratification approaches to the assessment of applications for registration of medicines.

Should SAHPRA wish to apply such risk-based approaches the following types of review may be considered (Green Thompson, 2008). The first is a full review of the complete quality, pre-clinical and clinical data applicable to medicines that have not been reviewed/approved by an NRA recognized by SAHPRA (Green Thompson, 2008). The second is an abridged review applicable to a medicine that has been reviewed/approved by one recognized NRA (World Health Assembly, 2014). Similar to the Mutual Recognition Procedure used in the European Union the abridged review makes use of the evaluation report and the regulatory decision of a recognized NRA to guide the evaluation of the medicine by SAHPRA (Green Thompson, 2008; Liberti, 2018). The third is the verification review that may be used to evaluate a medicine that has been approved by at least two recognized NRAs (Green Thompson, 2008). Through this review the product is validated for conformance to the authorized product specification (Pharasi and Banoo, 2015). The fourth is the evaluation of a dossier for a generic medicine (Green Thompson, 2008). The generic medicine should be approved by at least one recognized NRA and should correspond to the reference product (with the same dosage form and strength) registered by SAHPRA (Green Thompson, 2008).

Despite the type of review chosen for any given submission SAHPRA may insist that a full dossier consisting of complete quality, pre-clinical and clinical data is submitted upon application for medicine registration. Although a full assessment of the complete data may not be performed having the full dossier available on file will be advantageous for purposes of future reference or for post-market surveillance activities. A letter of intent to submit an application for registration of a medicine would be required to allow the regulator to adequately plan and allocate the necessary resources required to evaluate upcoming submissions. Through this process, the regulator may also anticipate whether specific expertise would be required in the assessment of the application and may be afforded the advantage of recruiting such expertise in advance thus circumventing unnecessary delays in the review process. This risk-based approach could be successfully applied provided that agreements are in place between SAHPRA and recognized NRAs to ensure that information pertaining to medicine assessment reports, post-marketing surveillance and post-marketing variations and/or amendments is easily shared and disclosed. As this system develops SAHPRA may consider introducing improved processes based on similar risk-stratification processes to address the submission of applications for variations and amendments to registered dossiers (Green Thompson, 2008). In re-designing the regulatory review process it would be prudent to consider the application of an appropriate framework for benefit-risk assessment to facilitate the evaluation of the benefit-risk balance of medicines prior to registration (Green Thompson, 2008; Leong et al., 2015). The implementation of GRPs and GRevP (South African Health Products Regulatory Authority [SAHPRA], 2016) and quality decision-making principles are also recommended with a view to reinforce transparent decision-making processes. Therefore, the application of risk-stratification approaches and FRPs would be an advantage when considered in line with the recommendations of the WHO (Ward, 2014; World Health Organization [WHO], 2014b). It remains to be seen if these proposed initiatives will be implemented by the new organization.

### Monitoring and Measuring

**FIGURE 4:**
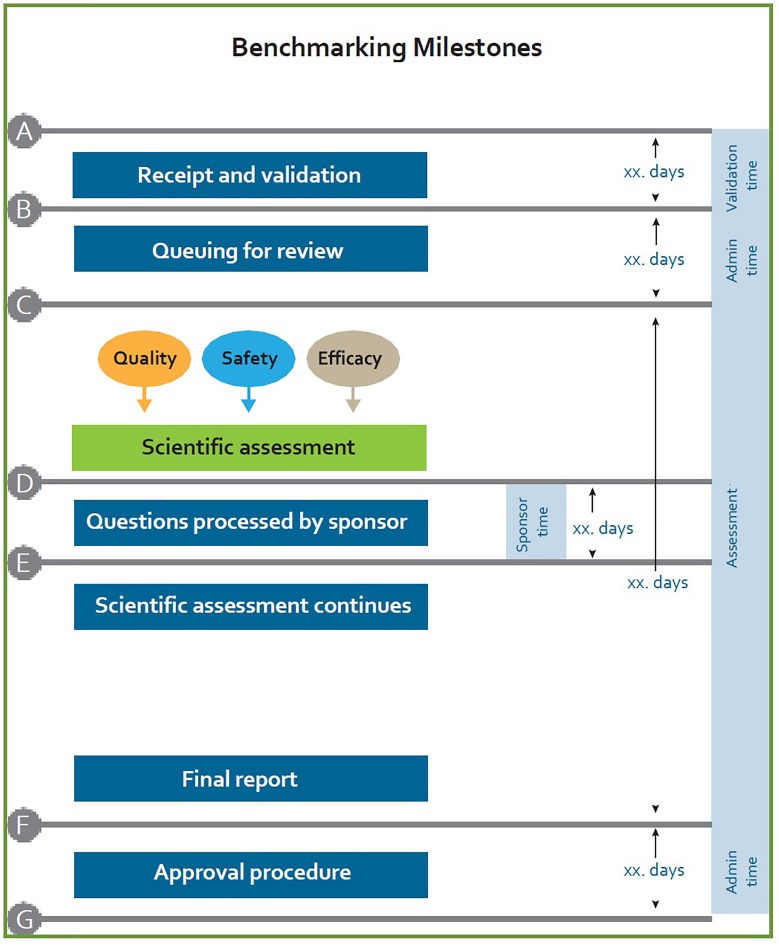
Benchmarking milestones currently utilized by regulatory authorities.

In the current model there is no target for overall approval time of applications for registration and no targets for the key review milestones. The targets for overall approval time and key review milestones need to be identified, codified into policy and guidelines, recorded, measured and monitored. Figure 4 provides a generic figure of individual milestones that have been used by other regulatory authorities and that may be considered for use within SAHPRA.

Appropriate systems and resources need to be put in place to support the accurate tracking of the overall approval times and key milestones in the regulatory review process. Administrative and technical screening time, queuing time prior to review and clock stops, measuring the time with applicants, must be recorded and monitored. The metrics collection process must be strengthened in order to allow measurement and improvement of SAHPRA regulatory performance.

With accountability and transparency being a focus within the medicine regulatory landscape in South Africa, SAHPRA has to be cognizant of the past administrative injustices and take ownership of its performance. SAHPRA targets for regulatory review must be communicated to all stakeholders and it must be held responsible for meeting its obligations in terms of such targets and demonstrate accountability to parliament, to the public, to the industry and to all relevant stakeholders (Green Thompson, 2008). Furthermore, SAHPRA should undertake to employ the basic principles of administrative justice within the routine practices and activities of the Authority (Green Thompson, 2008). Providing written reasons to support regulatory decisions made by the Authority could be one such practice that may support legal certainty and contribute to enhanced regulatory efficiencies and transparency (Green Thompson, 2008). *Quid pro quo* provisions to relieve applicants of consequences of regulatory under-performance may also need to be considered (Green Thompson, 2008).

## Key Recommendations for a New Regulatory Environment

In order to ensure the full potential of the new regulatory environment in South Africa the following recommendations are considered to be fundamental in underpinning the success of SAHPRA:

### Quality Management System

Establishment of such a system would help to safeguard accountability, consistency and transparency of SAHPRA and streamline the implementation of GRP and GRevP including quality decision-making practices and benefit-risk assessment.

### Measuring and Monitoring

This will ensure the measurement and improvement of regulatory performance, targets for overall approval time and key review milestones. Consequently, this will lead to the implementation of appropriate systems for and a culture of accurate metrics collection and measurement of key performance indicators and their continuous improvement.

### Risk-Based Approach to the Evaluation of Medical Products

This will help to implement the appropriate allocation of resources, codify the use of FRPs in policy and culture, apply a risk-based approach commensurate with the product’s risk to patients and apply increased resources for pharmacovigilance activities and to support the reliance and recognition of reference agencies.

### Training and Skills Development of Regulatory Expert Reviewers

Under the new SAHPRA initiative the review will be carried out by internal staff and as such there needs to be a structured program for the provision of ongoing training and skills development of regulatory reviewers (scientists, pharmacists, pharmacologists, and medical doctors).

The SAHPRA backlog clearance strategy requires that additional capacity and efforts have been made to recruit experienced reviewers to assist in this regard. There may be much value added in considering the possibility of establishing concomitant mentorship programs through which internal staff may benefit from the proposed “apprentice-orientated” learning opportunities that exist through the rollout of the backlog clearance strategy.

### Establishment of an Expert Advisory Committee

By establishing an expert advisory committee, this could be of value to support the regulatory reviewers as well as advise the industry on best practices and providing feedback on the quality of the submitted dossier.

## Conclusion

The purpose of this review was to provide insight into the history of the enabling legislation and expert reviews and recommendations for regulatory reform that have given rise to a new regulatory regime in South Africa. Many key opportunities and modalities for change have been identified and it is evident that re-enforcement of strategies to address inadequate financial and human resources, stakeholder relationships, paper-driven document management systems, service delivery and regulatory review processes, need to be considered in order to strengthen the regulatory systems in South Africa. With time and active leadership from the SAHPRA Board together with the SAHPRA CEO and the management team it is hoped that the re-engineered strategies and processes, planned for implementation will serve to enhance the regulatory landscape in South Africa.

## Author Contributions

AK, SB, SS, and SW contributed to the design and implementation of the research, to the analysis of the results and to the writing of the manuscript.

## Conflict of Interest Statement

The authors declare that the research was conducted in the absence of any commercial or financial relationships that could be construed as a potential conflict of interest. The reviewer CP and handling Editor declared their shared affiliation.

## Abbreviations

CEO: Chief Executive Officer
EDMS: electronic document management system
FRPs: facilitated regulatory pathways
GMP: good manufacturing practice
GRevP: good review practice
GRP: good regulatory practice
ICT: Information and Communication Technology
MCC: Medicines Control Council
NCEs: new chemical entities
NRAs: national regulatory authorities
PFMA: Public Finance Management Act
PMA: Pharmaceutical Manufacturers Association
QMS: quality management system
SAHPRA: South African Health Products Regulatory Authority
SAMMDRA: South African Medicines and Medical Devices Regulatory Authority
TORS: Technical Operations and Regulatory Strategy
WHO: World Health Organization.
